# Incomplete Region Estimation and Restoration of 3D Point Cloud Human Face Datasets

**DOI:** 10.3390/s22030723

**Published:** 2022-01-18

**Authors:** Kutub Uddin, Tae Hyun Jeong, Byung Tae Oh

**Affiliations:** School of Electronics and Information Engineering, Korea Aerospace University, Goyang 10540, Korea; md.ku.ud.ra@gmail.com (K.U.); sdfds5566@kau.kr (T.H.J.)

**Keywords:** 3D point cloud, incomplete region, deep learning, estimation, and restoration

## Abstract

Owing to imperfect scans, occlusions, low reflectance of the scanned surface, and packet loss, there may be several incomplete regions in the 3D point cloud dataset. These missing regions can degrade the performance of recognition, classification, segmentation, or upsampling methods in point cloud datasets. In this study, we propose a new approach to estimate the incomplete regions of 3D point cloud human face datasets using the masking method. First, we perform some preprocessing on the input point cloud, such as rotation in the left and right angles. Then, we project the preprocessed point cloud onto a 2D surface and generate masks. Finally, we interpolate the 2D projection and the mask to produce the estimated point cloud. We also designed a deep learning model to restore the estimated point cloud to improve its quality. We use chamfer distance (CD) and hausdorff distance (HD) to evaluate the proposed method on our own human face and large-scale facial model (LSFM) datasets. The proposed method achieves an average CD and HD results of 1.30 and 21.46 for our own and 1.35 and 9.08 for the LSFM datasets, respectively. The proposed method shows better results than the existing methods.

## 1. Introduction

The massive advancement in modern technology, especially in 3D scanners such as LiDAR (light detection and ranging), allows us to capture datasets in a more convenient format (3D point cloud) to represent 3D objects. The 3D point cloud dataset contains geometric information of objects along the *x*, *y* and *z*-axes. Sometimes, it may also contain normal and color information of the objects. Presently, the use of 3D point cloud datasets is increasing significantly owing to the multitude of applications, such as recognition [1,2,3,4], classification, segmentation [5,6,7,8,9], and upsampling [10,11,12,13]. The recognition, classification, segmentation, and upsampling performance depend significantly on the quality of the 3D point cloud datasets.

However, an incomplete dataset degrades the performance of the detection, classification, segmentation, and upsampling methods mentioned above. There are several reasons for incomplete regions in the acquisition of 3D point cloud datasets, such as scanner placement, occlusions, low reflectance of the scanned surface, viewing directions, and packet loss [14].

**Figure 1:**
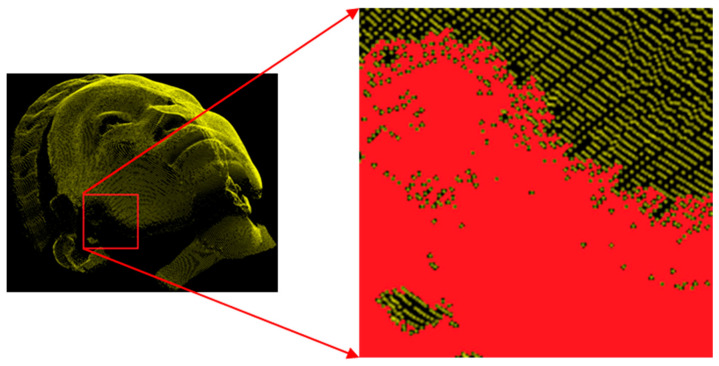
An example of incomplete regions indicated with red whereas the complete region’s points presented with yellow.

An example of incomplete regions in the 3D point cloud human face data is shown in Figure 1, where the red regions indicate the incomplete regions. For applications ranging from recognition to upsampling, estimating incomplete regions in the point cloud is vastly an important task to provide a high-quality 3D model. Recently, several studies were conducted to fill in missing points in the 3D point clouds. Most of the methods focus on the information about the distribution and curvature of the neighboring points as well as the mesh information. However, when the incomplete regions are large and have a complex surface structure, such as the human face, it becomes difficult to estimate the missing points.

In this study, we propose a new method to estimate the incomplete regions of the 3D point cloud human face datasets. The proposed method can estimate any kind of incomplete regions in the 3D human face point cloud datasets, particularly those caused by scanner placement, viewing direction, and packet loss. The estimated points of the incomplete regions have a different distribution from the complete regions. Therefore, we restored the estimated point cloud to improve its quality. The main contributions of this study can be summarized as follows.

1. First, we perform preprocessing, such as rotation along the left and right angles of the input point cloud. From the rotated view, we generate a mask and project it onto the 2D surface. The generated mask and the 2D projection are interpolated to propagate the interpolated output. Then, we perform reconstruction to generate the estimated point cloud.
2. To improve the quality of the estimated regions, we apply a deep learning model to restore the point cloud. We consider a dynamic graph convolutional neural network (DGCNN) that introduces edge convolution to accomplish the restoration.
3. We evaluated the proposed method on our own and LSFM datasets to show the robustness of the proposed system. The proposed method can considerably improve the quality of the estimated point cloud.
4. We also compared the proposed method with state-of-the-art point cloud processing systems to show the effectiveness and robustness. The proposed method achieved better results than previous methods.

The rest of this paper is stated as follows. We demonstrate the related works in Section 2. In Section 3, we concentrate on the illustration of the proposed method including architectural overviews, incomplete region estimation and deep learning-based restoration. We describe the environmental conditions, performance evaluations and comparisons in Section 4. Finally, in Section 5, we consolidate the discussion and present conclusions.

## 2. Related Works

The estimation of missing points in the 3D point cloud is of great significance for the development of point cloud-based applications, especially for human face datasets. In recent years, many methods were suggested filling the incomplete regions ranging from polygon mesh [15,16,17,18,19,20,21] to the 3D point cloud. Filling holes in polygon mesh have been widely conducted for geometric modeling, computer graphics and image processing considering various deficiencies such as local connectivity, global topology and geometry. Li et al. [16] searched the feature points from the neighboring points of the hole and built a polynomial blending curve to fill the missing parts. In [17,18,19], the hole parts of the triangular mesh were filled based on surface fitting using the neighboring points. Enkhbayar et al. [20] proposed filling complex holes in the 3D mesh by inserting locally uniform points into the hole created by contour curves. Many methods have been proposed to fill the holes in the 3D point cloud, which are constrained by various factors such as geometric structure and size of incomplete regions.

The concepts used to fill the polygon mesh are accelerated to fill missing points in the 3D point cloud [21,22,23,24,25,26,27,28,29,30,31,32]. Several methods introduced the estimation of incomplete regions in the point cloud, which can be divided into mesh-based [21,22,23,24,25] and direct point cloud-based estimation [26,27,28,29,30,31,32].

The mesh-based hole-filling methods conduct two steps: triangulation and surface reconstruction. Wang et al. [21] created a triangular mesh and identified the points adjacent to the holes. Then, the moving least squares method was used to estimate the missing points. In [18,22], the incomplete regions were filled using triangulation methods in which the surface was constructed by a surface fitting function. Nguyen et al. [23] proposed a triangular mesh-based method to fill concave regions. First, they triangulated the 3D point cloud into a 3D grid and separated the incomplete regions. Then, the boundaries of the holes were determined and new points were inserted into the holes. Furthermore, triangulation was done to generate the final point cloud. Nguyen et al. [24] introduced the method for incomplete regions filling based on the computation of tangent plane for the boundary point of holes. First, they extracted the exterior boundary of a point cloud on a projected 2D grid. Then, they identified the hole boundary and filled the holes based on the tangent plane for each boundary point. Quinsat et al. [25] described a mesh deformation method for completing digitized holes in the 3D point cloud. They accomplished the nominal mesh deformation by determining the difference between the nominal mesh and the point cloud.

The efficiency of mesh-based methods for filling holes in the 3D point clouds strongly depends on the mesh structure. The better the quality of the mesh, the better the performance. In addition, it becomes very difficult to fill the holes correctly if the number of points is too large and the structure is complicated. Considering the above drawbacks, there are very few methods that have illustrated to fill the missing points directly from the 3D point cloud. Chalmoviansk et al. [26] used adjacent points around the hole boundary and constructed a surface to fill the incomplete regions. Qiu et al. [27] presented a 3D point cloud hole-filling approach by describing a certain relationship between the incomplete parts and the surface surrounding them. They determined the local patches of points with neighboring points of holes and filled the holes with the points on the patches. Chen et al. [28] introduced a radial basis function to interpolate the boundary points and then filled the hole regions uniformly. In [29], Wu et al. proposed a hole-filling method based on the boundary extension and convergence. First, boundary points were moved to the hole regions and the correlation between the neighboring points were increased by applying a smoothing operation. Lin et al. [30] developed a featured curves-based to fill the hole regions directly from the point cloud. They identified the boundary points and the feature points of the hole to categorize the feature and the non-feature points. Finally, the hole regions were subdivided into sub-holes and filled with tensor voting. Dinesh et al. [31] provided an exemplar-based framework for filling holes by exploiting non-local self-similarity. Wang et al. [32] used feature lines such as hole boundary lines, ridge lines and valley lines to generate curves and fitted them to fill the holes.

Completing holes directly from the point cloud does not require transforming the point cloud to the mesh. Therefore, this method is much faster than the mesh-based estimation. The performance of these methods depends on the data size and the structure of the 3D object. When the data size and structure of the objects become large and complicated, the above method cannot fill the incomplete regions effectively and efficiently.

To address the above drawbacks of the mesh-based and direct point cloud-based hole-filling methods, we perform the estimation of points for incomplete regions using the masking method and the restoration of the estimated points using deep learning to make them resemble the complete regions.

To restore the estimated point cloud, we conduct the DGCNN model which is explained in Section 3. We also use the point cloud upsampling network (PU-Net) [10] and point cloud upsampling generative adversarial network (PU-GAN) [11] for comparison with the proposed method. PUNet and PU-GAN had been used for upsampling the point cloud. However, we use these models for restoration purposes. To compare the proposed method with a more recent point cloud processing technique, we use a point cloud denoizing method called differential manifold reconstruction for denoizing (DRMD) [33] which is more appropriate for the restoration of the estimated point cloud. In this method, Luo et al. tried to remove the noise to improve the quality of the noisy point cloud. They applied differential pooling, manifold reconstruction and resampling to generate denoized point clouds.

## 3. Proposed Methodology

This section covers the major contents of the proposed method, including an architectural overview, point cloud estimation, and restoration of incomplete regions.

### 3.1. Architectural Overview of the Proposed Method

**Figure 2:**
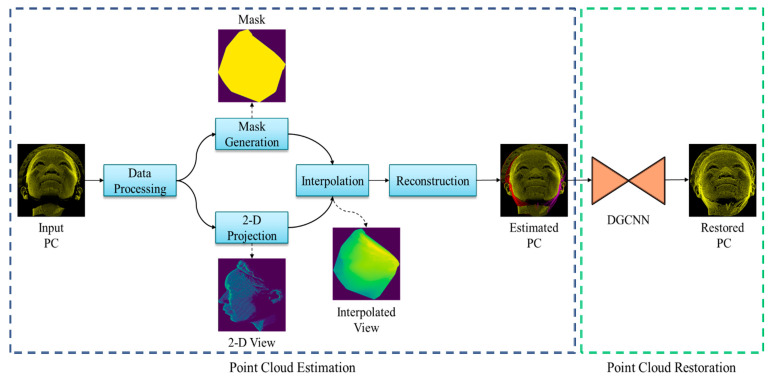
Block diagram of the proposed method including point cloud estimation and restoration modules.

A block diagram of the proposed system is depicted in Figure 2. The overall architecture consists of two major parts: (1) point cloud estimation and (2) point cloud restoration. The input point cloud (front view) is passed through the estimation module, which performs several operations to fill out the incomplete regions. The estimated points (front view with left and right estimation) were then restored using a deep learning model. The details of point cloud estimation and restoration are illustrated below.

### 3.2. Point Cloud Estimation of Incomplete Regions

The configurations of the device, environmental factors and other settings can cause incomplete regions in the point cloud dataset. These missing regions can degrade the quality of the 3D point cloud dataset, which can affect the performance of the point cloud processing system. The input point cloud (front view) has a very small number of points than the ground truth point cloud due to incomplete regions. Therefore, we cannot directly apply point cloud processing to the front view point cloud using deep learning. As a result, it is very important to fill the incomplete regions in an effective and efficient way for further processing, for example, point cloud restoration. In this section, we present a simple model-based approach to estimate the missing points for incomplete regions so that we can employ the proposed deep learning model for restoration.

Let us consider $P_{f}$ as the input point (front view) cloud of size N×3. Then, $P_{f}$ is rotated along the x-axis in the right and left sides, defined as follows:

(1) $$P_{\theta }=\varphi \left(P_{f};\;\theta \right)\;$$

where φ(.) represents the rotation function with angle θ and $P_{\theta }$ is the rotated point cloud.

From the rotated point cloud, we first generate mask $M_{\theta }$ to separate the foreground and background regions and then obtain its projected 2D image, $I_{P}=f_{P}\left(\;P_{\theta }\right)$. From multiple 2D projected images, we apply conventional interpolation method such as bicubic interpolation to estimate the fully complete point cloud $P_{e}$, which can be mathematically written as follows:

(2) $$P_{e}=f_{I}\left(P_{\theta };\left\{I_{P}\right\},\left\{M_{\theta }\right\}\right)\;$$

where $f_{I}\left(.\right)$ indicates an image-based interpolation scheme. We have used the bicubic method in this study [34]. The esimated points are not sufficiently accurate, which is further restored by the subsequent restoration model.

### 3.3. Deep Learning-Based Point Cloud Restoration

The significant improvements in machine learning, particularly deep learning, allows us to easily perform the detection, recognition, classification and restoration or reconstruction of data for various purposes. Point cloud restoration is one of the most important tasks for other applications such as object detection and face recognition.

Compared to traditional deep learning models, such as convolutional neural network, recurrent neural network and long short-term memory, the DGCNN model works better in point cloud processing [9]. Therefore, we design a DGCNN model to restore the estimated point cloud, which was applied for point cloud classification and segmentation. Traditional point cloud processing models, such as PointNet [5] and PointNet++ [6] operate on individual points independently to explore the local information and maintain permutation invariance. These models ignore the geometric relationship among the neighboring points. However, DGCNN applies convolution-like operations to address the geometric relationship among the edges of neighboring points called edge convolution (EdgeConv). EdgeConv extracts edge features between a point and its adjacents. It is independent of the ordering of points that makes it permutation invariant. The DGCNN is dynamically updated after each layer, instead of being fixed in GCNN [13].

#### 3.3.1. Architecture of the Proposed DGCNN Model

**Figure 3:**
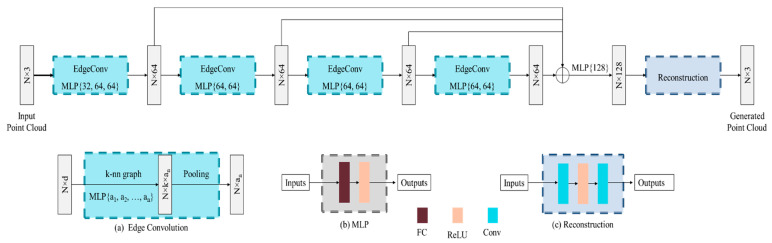
Architecture of the proposed DGCNN for the restoration of estimated point cloud.

The architecture of the DGCNN model is shown in Figure 3. The proposed DGCNN model consists of four EdgeConv layers. The first EdgeConv layer is composed of three multilayer perceptron (MLP) networks (32, 64 and 64 output features) and the remaining three EdgeConv have two MLP networks (64 and 64 output features).

**Figure 4:**
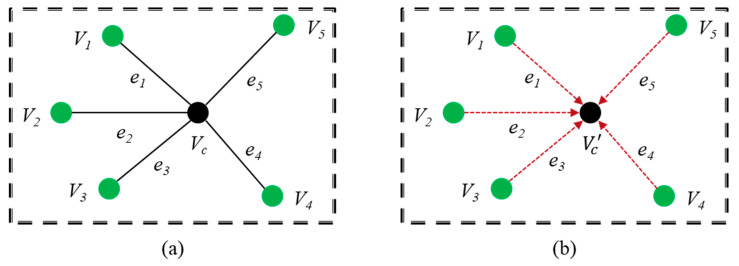
Edge convolution; (**a**) edges with associated vertices and (**b**) scenarios of edge convolution.

The EdgeConv layer computes the edge features for each point, as shown in Figure 4, where $V_{i}$ and $e_{j}$ (i, j=1, 2, 3, 4, 5) indicate the vertices and edges. The $V_{c}$ and $V_{c}^{’}$ represent the center vertex and output of the edge convolution, respectively.

Let us consider a point cloud $P=\left\{P_{1},\;P_{2},\;\ldots ,\;P_{N}\right\}$ with *N* points along the three axes *x*, *y* and *z*, respectively. We can represent the point cloud P as a graph, G=(V, e) where V and e are the vertices and edges. We construct G as the k-nearest neighbor (k-NN) graph of P for *N* points along the three axes. Then, the edge features are computed as follows:

(3) $$e_{ij}=h_{\Theta }\left(P_{i},\;P_{j}\right)\;$$

where $h_{\Theta }$ is a non-linear function with a set of learnable parameters Θ. Finally, the EdgeConv is accomplished by applying channel-wise symmetric aggregation on the edge features defined as follows:

(4) $$V_{c}^{\prime }=\underset{j:\left(i,j\right)\in \varepsilon }{□}h_{\Theta }\left(P_{i},\;P_{j}\right)\;\;$$

where the choice of edge function, *h* and aggregation, □ are described in detail [9].

Each MLP layer consists of one fully connected and one rectified linear unit (ReLU) layer. The fully connected layer learns the non-linear combination of features. The ReLU is used to consider only the positive response. After the MLP operations, we apply a pooling layer among the adjacent features to generate an $N\times a_{n}$ dimensional feature from an $N\times k\times a_{n}$ dimensional feature as shown in Figure 3a. Here, N, k and $a_{n}$ indicate the number of input points, the number of neighboring points and the number of MLP layers, respectively. We have fixed the value of k as described in [9]. Each EdgeConv layer generates 512×64 dimensional features which are then concatenated and passed a 512×256 features map through a MLP layer again. The MLP layer reduces the size of features map from 512×256 to 512×128. We additionally integrate a reconstruction layer that conducts convolution, ReLU and convolution operations to generate the final point clouds. The first convolution operation generates to 512×64 dimensional features and passes through a ReLU operation. Finally, the second convolution produces the final outputs of shape 512×3.

#### 3.3.2. Loss Function for the Point Cloud Restoration

We design a loss function by assembling two types of losses: CD [11] and repulsion loss (RL) to train the restoration network. Since the ground truth and predicted point clouds are unordered, we use permutation invariant symmetric CD loss. The CD measures the similarity between the ground truth point cloud ($P_{gt}$) and the predicted point cloud ($P_{pd}$). For given $P_{gt}$ and $P_{pd}$ with N points, CD considers the distance of each point in $P_{gt}$ and finds nearest point in $P_{pd}$. Then, it calculates the sums of the square distance from $P_{gt}$ to $P_{pd}$ and vice-versa which can be defined as follows:

(5) $$L_{CD}\left(P_{gt},\;P_{pd}\right)=\frac{1}{\left|P_{gt}\right|}\underset{x\in P_{gt}}{\sum }\underset{x^{\prime }\in P_{pd}}{\min}{\left|\left|x-x^{\prime }\right|\right|}^{2}\;+\frac{1}{\left|P_{pd}\right|}\underset{x\in P_{pd}}{\sum }\underset{x^{\prime }\in P_{gt}}{\min}{\left|\left|x-x^{\prime }\right|\right|}^{2}\;\;\;\;\;\;\;\;$$

where first and second parts ensure that $P_{pd}$ accurately aligns to $P_{gt}$ and $P_{gt}$ is accurately aligned to $P_{pd}$, respectively.

RL is used to make the distribution of the predicted points resemble to the original points. RL can be defined as follows:

(6) $$L_{RL}=\overset{N}{\underset{i=0}{\sum }}\underset{i^{\prime }\in K\left(i\right)}{\sum }\eta \left(\left|\left|p_{i}^{\prime }-p_{i}\right|\right|\right)w\left(\left|\left|p_{i}^{’}-p_{i}\right|\right|\right)\;\;\;\;\;$$

where K(i) indicates the indices of the neighboring points of $p_{i}$; η(.) and w(.) represents the repulsion terms and the fast-decaying weight function, as described in [10].

Then the DGCNN is trained by minimizing the following joint loss of $L_{CD}$ and $L_{RL}$.

(7) $$L_{total}=\alpha L_{CD}+\beta L_{RL}$$

where α and β are the balancing parameters of $L_{CD}$ and $L_{RL}$ losses.

## 4. Experimental Results

In this section, we present the detailed experiments of the proposed method including the environmental setups, evaluation metrics, performance evaluations and comparisons and complexity analysis.

### 4.1. Environmental Setups

We ran the entire experiment on a Linux 20.04 (Ubuntu) operating system with a GPU named GeForece GTX 1080. Python was used as the programming language. We mainly used the PyTorch framework with torch geometric to design the DGCNN model for the restoration of the estimated point cloud. We performed the experiments on our own 3D point cloud human face and LSFM datasets [35].

Our own dataset contains ten samples collected by ten individuals. The dataset is collected using the Onscans IU-50E device [36]. Each sample has an average of 100 K points. Initially, we have ten ground truth samples that contain merge view point clouds including front, left and right view point clouds. First, we project the merge view point cloud onto the 2D space and generate the front view dataset. The front view dataset has missing points on the left and right sides. We used the proposed estimation method to estimate the missing regions on the left and right sides. The estimated and ground truth datasets are different. As a result, we need to restore the estimated point cloud. We performed the patch-based restoration owing to the limited amount of data and computational efficiency. Therefore, we split the points into patches of size 512 × 3 for training the DGCNN model. We randomly selected 1000 patches for each sample. We used the first four samples as test samples and the remaining six samples were used for training.

The LSFM dataset contains 10 K individuals of human face 3D point cloud data in which each class has 50 data. We took 1000 samples from separate individuals for our experiments. Each sample contains 28,588 points. Since we performed the patch-based restoration, we split the dataset into patches of size 512 × 3. We used the first four hundred samples as a test set and the remaining six hundred samples as a training set.

To train the DGCNN, we set the batch size to 64 and the training process was continued up to 500 epochs. We used the Adam optimizer with a learning rate of 0.001 and $\beta_{1}$, $\beta_{2}$ = (0.9, 0.999) for optimization. Adam optimizer is used to update the weights iteratively during the training. It combines the advantages of adaptive gradient descent (AdaGrad) and root mean square propagation (RMSProp). It is also faster than AdaGrad, RMSProp and stochastic gradient descent. The learning rate decayed after every 100 epochs with a factor of 0.8.

### 4.2. Evaluation Metrics

We evaluated the proposed estimation and restoration methods by calculating CD and HD [37]. We have already discussed about CD in Section 3.3.2. HD is used to determine the similarity between two-point clouds. It overcomes the problems caused by different grid sizes, highly overlapping and very large-scale point sets.

Let us consider $P_{e}$ and $P_{r}$ as the input (estimated) and restored point cloud, respectively. Then, HD is obtained from the maximum one-sided HD (OHD) between $P_{e}$ and $P_{r}$ and between $P_{r}$ and $P_{e}$ defined as follows:

(8) $$HD\left(P_{e},\;P_{r}\right)=max\left\{OHD\left(P_{e},\;P_{r}\right),\;OHD\left(P_{r},\;P_{e}\right)\right\}$$

For $P_{e}$ and $P_{r}$, the $OHD\left(P_{e},\;P_{r}\right)$ can be formulated as follows:

(9) $$OHD\left(P_{e},\;P_{r}\right)=\frac{1}{V}\underset{x_{i}\in P_{e}}{\max}\{\underset{j_{j}\in P_{r}}{\min}||x_{i}-y_{j}|{|}^{2}\}$$

where V is the volume of the parallelepiped for a given point cloud. A lower HD value indicates a smaller gap between the input and restored point clouds.

### 4.3. Performance Evaluations and State-of-the-Arts Comparsons

**Figure 5:**
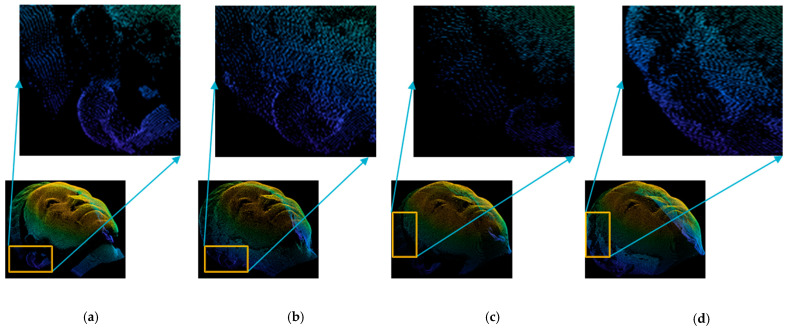
Point cloud estimation of incomplete regions from front view in our own dataset. (**a**) Sample-1 (front view). (**b**) Sample-1 (estimation). (**c**) Sample-2 (front view). (**d**) Sample-2 (estimation).

First, we evaluated the performance of the proposed method on our own dataset. We estimated the missing points in the incomplete regions by performing preprocessing as described in Section 3.2. For better understanding, we provided two samples of the front view and the estimated point cloud, as visualized in Figure 5. As shown in Figure 5b,d, the empty space of the front view point cloud is effectively filled by the proposed method, which is much better than the incomplete front views, as given in Figure 5a,c.

The estimated point cloud is not sufficient compared to the ground truth point cloud. To improve the quality of the estimated point cloud, we integrated the restoration process after the estimation. We used four test samples to show the experimental results of the proposed restoration method. We have separately reported the results of CD and HD for each test sample for ground truth versus estimation, ground truth versus restoration and estimation versus restoration.

Table 1 shows CD and HD results of four test samples for the ground truth versus estimated and ground truth versus restoration. Initially, we reported the results between the ground truth and the proposed estimated data as given at first row in Table 1. The average results of CD and HD are 2.37 and 22.82, approximately. After obtaining the estimated point cloud, we applied the proposed DGCNN model to restore them. The proposed method secures average CD and HD results of about 1.30 and 21.46, which are much better than the estimated results. We compared the restoration results of the DGCNN with PU-Net, PU-GAN and DRMD methods. It is evident that the restoration with PU-Net degrades the quality of the estimated point cloud and obtains CD and HD results of 9.65 and 25.52, respectively. PU-GAN and DRMD can effectively decrease CD (2.17 and 2.52) and HD (21.64 and 22.20) results, which is better than both proposed estimation and PU-Net. However, the proposed DGCNN model outperforms not only the estimation but also restoration using PU-Net, PU-GAN and DRMD, respectively.

**Figure 6:**
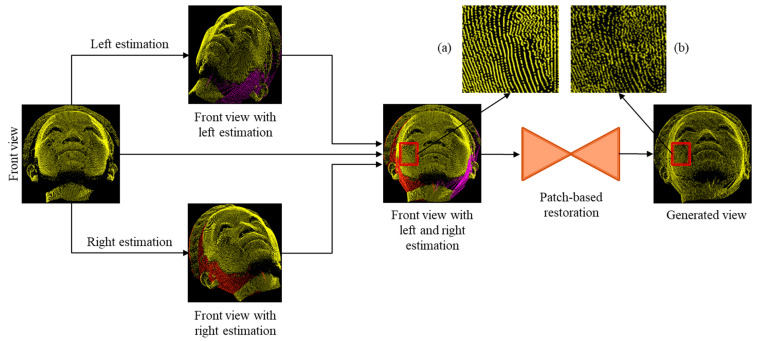
An example of incomplete region estimation and restoration of our own 3D point cloud human face dataset.

An example of the overall process is shown in Figure 6, in which the front view input point cloud is passed through the left and right estimation modules to fill the incomplete regions on the left and right sides indicated by purple and red colors. The front view, left estimation and right estimation are then combined to generate an estimated point cloud with filled incomplete regions. A restoration model is then integrated to restore the estimated point cloud to make them resemble the ground truth point cloud. To compare the visual results, we added two patches from the estimated and restored point clouds, as given in Figure 6a,b.

**Figure 7:**
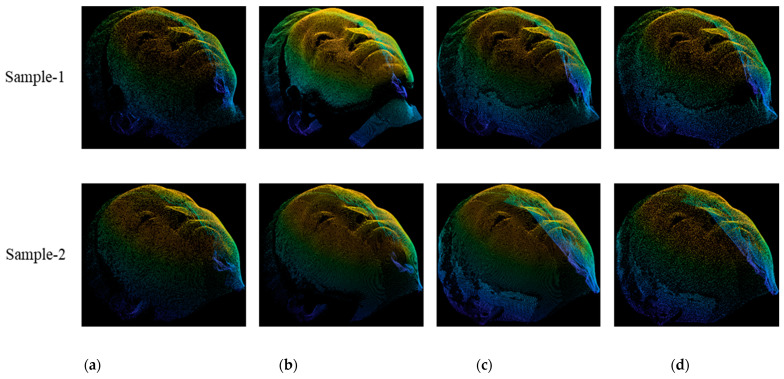
Comparison of point cloud among (**a**) ground truth, (**b**) front view, (**c**) estimation and (**d**) restoration in our own dataset.

We provided the visual comparisons among the points clouds, as shown in Figure 7. Figure 7a–d present the ground truth, front view, estimation and restoration point clouds. It can be observed that the estimated point cloud is filled correctly and denser than the front view and ground truth point cloud. Therefore, we apply restoration to generate the points that are much closer to the ground truth compared with estimation.

**Figure 8:**
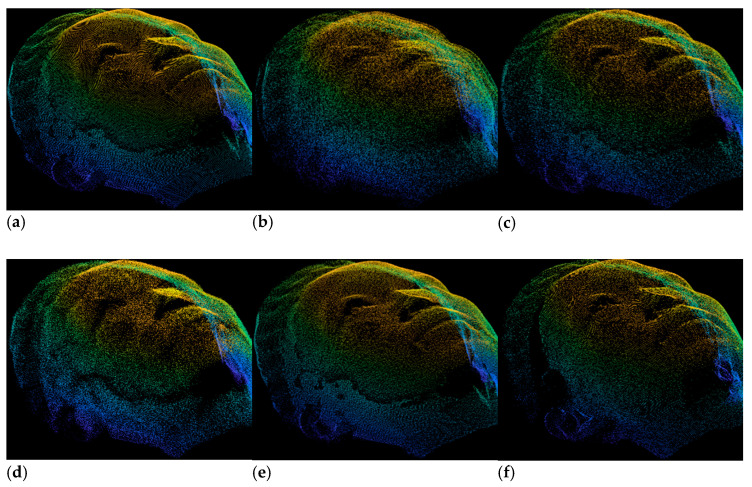
Visual comparisons of restoration of our own 3D point cloud dataset. (**a**) Estimation. (**b**) PU-Net [10]. (**c**) PU-GAN [11]. (**d**) DRMD [34]. (**e**) Proposed. (**f**) Ground Truth.

For better understanding and comparison, we also applied the restoration with our proposed DGCNN and compared it with PU-Net, PU-GAN and DRMD methods. Figure 8a–f depict the estimated, restored with PU-Net, PU-GAN, DRMD, DGCNN and ground truth point clouds, respectively. From Figure 8, it can be noticed that the visual result of the proposed DGCNN is much better than the results obtained by estimation, PU-Net, PU-GAN and DRMD, respectively. In Figure 8b, we can also notice that the restored points obtained by PU-Net and DRMD are too noisy and completely misaligned compared to estimated and ground truth point clouds. PU-GAN can restore the estimated point which is better than PU-Net and DRMD but less than the proposed DGCNN model. Therefore, we can emphasize that the proposed DGCNN can effectively restore the estimated point cloud of our own dataset than the prior point cloud processing techniques.

We also evaluated the performance on LSFM dataset for showing the generalization ability and robustness of the proposed method. Table 2 list the average CD and HD results for test set in LSFM dataset. Similar to our own 3D point cloud human face dataset, the proposed DGCNN model shows better CD and HD results for ground truth versus restoration than the prior works. The proposed method assures an average CD result of 1.35 for ground truth versus restoration which is better than the proposed estimation (2.59), PU-Net (8.81), PU-GAN (1.53) and DRMD (2.32), respectively. The average HD result of 9.08 obtained by the proposed restoration model is also better than the proposed estimation (14.41), PU-Net (16.68) and DRMD (11.39), but less than PU-GAN (8.38) in the LSFM dataset.

**Figure 9:**
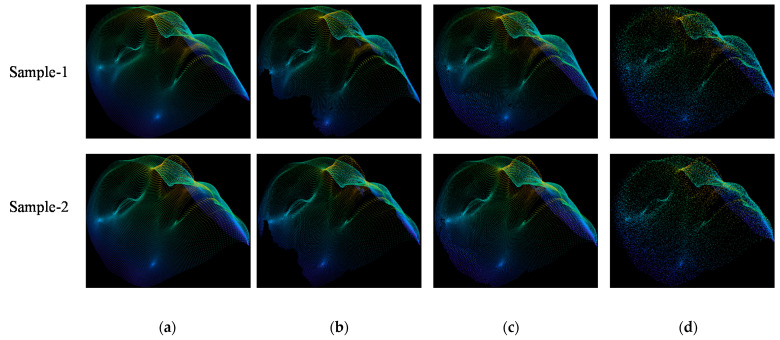
Comparison of point cloud among (**a**) ground truth, (**b**) front view, (**c**) estimation and (**d**) restoration in LSFM dataset.

Figure 9 visualizes two samples of the ground truth, front view, estimation and restored data in LSFM dataset. The front view data as shown in Figure 9b contains large incomplete regions which is estimated by the proposed method as shown in Figure 9c. The points in estimated regions are different from the complete regions. To make them resemble to ground truth, we applied restoration as shown in Figure 9d.

**Figure 10:**
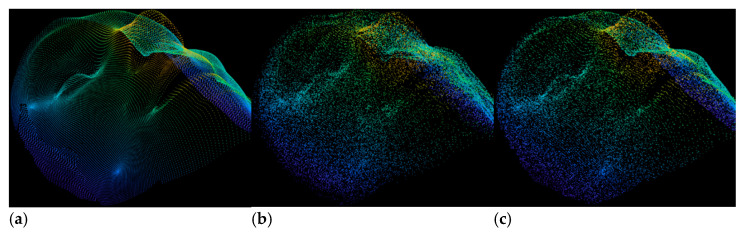

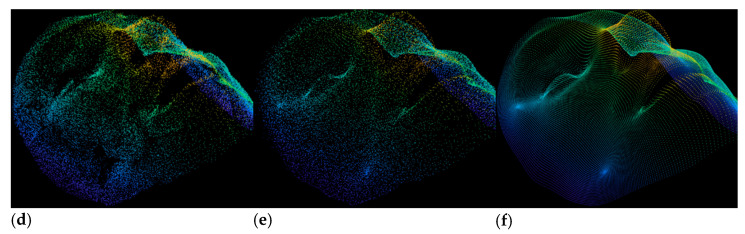
Visual comparisons of restoration of our own 3D point cloud dataset. (**a**) Estimation. (**b**) PU-Net [10]. (**c**) PU-GAN [11]. (**d**) DRMD [34]. (**e**) Proposed. (**f**) Ground Truth.

To provide better comparisons, we visualized the restored point clouds obtained by PU-Net, PU-GAN, DRMD and DGCNN in the LSFM dataset as shown in Figure 10. The restored point cloud using PU-Net and DRMD are much worse as shown in Figure 10b,d. However, PU-GAN and proposed DGCNN can effectively restored the estimated points as shown in Figure 10c,e.

From the experimental results and above discussions, we can say that the proposed method including incomplete regions estimation and restoration of 3D human face point cloud datasets, provides better performance than the prior point cloud processing techniques. We evaluated the proposed method on two different datasets and compared the results with three-point cloud processing techniques. The proposed method always shows better performance and can significantly improve the quality of the incomplete point cloud than the prior works. Therefore, we can assure that the proposed method is much robust than the prior works.

### 4.4. Complexity Analysis

We determined the complexity of the model in terms of parameters, floating-point operations per second (FLOPs) and runtime. For better understanding and explanation, we reported the details about the network complexity. Table 3 lists the number of parameters in each layer with the corresponding layer type and output shape. Table 4 provides the total number of parameters in millions, FLOPs in giga and time in seconds. We computed the number of parameters and FLOPs for a patch. The proposed DGCNN model has very less parameters and performed smaller number floating-point operations than the state-of-the-art methods.

We calculated the average time required to restore the test samples. The average test time for each sample in our own dataset is four times larger than the LSFM dataset. It is because each sample in our own dataset contains about 100 K points which is approximately four times larger than the number of points in LSFM dataset about 28 K. We compared the run time of the proposed method with the state-of-the-art methods as given in Table 4. The proposed method can restored the estimated points faster than DRMD but slower than PU-Net and PU-GAN, respectively.

## 5. Discussion and Conclusions

The major concern of the proposed method is to estimate the incomplete region by generating a mask and projecting onto the 2D surface of the input point cloud. The final estimation was performed by interpolating the mask and the 2D projection. The estimated points can fill in missing regions effectively. However, the distribution of the estimated point cloud is different from that of the ground truth point cloud. Sometimes, the estimated points of the incomplete regions can be denser than the points of the complete regions. This limits the proposed method and may decrease the performance of point cloud processing. Therefore, we conducted a deep learning model to restore the estimated points to the same extent as the ground truth points. We designed a DGCNN model that integrates multiple edge convolution blocks and a reconstruction block to restore the estimated points. We evaluated the performance of the proposed method on two datasets (our own and LSFM 3D point cloud human face datasets) to show the effectiveness. We also compared the results with PU-Net, PU-GAN and DRMD applied for restoring the estimated points. The proposed method improved the quality of the point cloud dataset of human faces with estimation and restoration.

## Figures and Tables

**Table 1:** Performance evaluation on our own dataset between ground truth and restoration.

Methods	Sample-1		Sample-2		Sample-3		Sample-4		Average	
	CD	HD	CD	HD	CD	HD	CD	HD	CD	HD
Proposed [Estimation]	2.38	20.52	2.22	20.83	2.31	13.19	2.55	36.73	2.37	22.82
PU-Net [10]	9.12	25.30	8.96	27.66	10.49	20.32	10.01	28.80	9.65	25.52
PU-GAN [11]	2.11	19.81	2.02	23.00	2.10	14.42	2.43	29.32	2.17	21.64
DRMD [33]	2.48	22.37	2.40	23.34	2.38	14.43	2.82	28.66	2.52	22.20
Proposed [Restoration]	1.37	19.82	1.22	21.97	1.11	14.58	1.51	29.47	1.30	21.46

**Table 2:** Performance evaluation on LSFM dataset between ground truth and restoration.

Methods	Proposed [Estimation]		PU-Net [10]		PU-GAN [11]		DRMD [33]		Proposed [Restoration]	
	CD	HD	CD	HD	CD	HD	CD	HD	CD	HD
Ground Truth versus Restoration	2.59	14.41	8.81	16.68	1.53	8.38	2.32	11.39	1.35	9.08

**Table 3:** Layers, output shape and parameters configuration in DGCNN model.

SL. No	Layer (Type)	Output Shape	Parameters
1	Linear, ReLU Linear, ReLU Linear, ReLU DynamEdgeConv	(32) (64) (64) (64)	224 2112 4160 -
2	Linear, ReLU Linear, ReLU DynamEdgeConv	(64) (64) (64)	8256 4160 -
3	Linear, ReLU Linear, ReLU DynamEdgeConv	(64) (64) (64)	8256 4160 -
4	Linear, ReLU Linear, ReLU DynamEdgeConv	(64) (64) (64)	8256 4160 -
5	Linear, ReLU	(128)	32,896
6	Conv1d, ReLU Conv1d	(64,512) (3512)	8256 195

**Table 4:** Model complexity.

Method	Parameters (M)	FLOPs (G)	Time (S)	
			Our	LSFM
PUNet [10]	0.814	1.48	15.50	8.46
PU-GAN [11]	0.516	0.80	24.16	10.68
DRMD [33]	0.225	0.62	57.61	20.13
Proposed	0.085	0.22	39.38	13.91
